# Severity of complications is associated with impaired health‐related quality of life in people with type 1 diabetes

**DOI:** 10.1111/dom.70306

**Published:** 2025-12-02

**Authors:** Sara Barraud, Gloria A. Aguayo, Emmanuel Cosson, Chloé Amouyal, Sylvie Feldman, Jean‐François Gautier, Patricia Vaduva, Samy Hadjadj, Hélène Hanaire, Laurence Kessler, Emmanuelle Lecornet‐Sokol, Pascale Massin, Louis Potier, Eric Renard, Yves Reznik, Agnès Sola, Anne Vambergue, Camille Vatier, Bruno Vergès, Jean‐Pierre Riveline, Guy Fagherazzi

**Affiliations:** ^1^ CRESTIC, Service d'Endocrinologie Diabète Nutrition URCA, CHU de Reims Reims France; ^2^ Deep Digital Phenotyping Research Unit, Department of Population Health Luxembourg Institute of Health Strassen Luxembourg; ^3^ Service d'Endocrinologie‐Diabétologie‐Nutrition AP‐HP, Avicenne Hospital, Paris 13 University, CRNH‐IdF, CINFO, Université Sorbonne Paris Nord Bobigny France; ^4^ Equipe de Recherche en Epidémiologie Nutritionnelle (EREN), Inserm (U1153) Université Paris 13, COMUE Sorbonne‐Paris‐Cité, Inra (U1125), Centre d'Epidémiologie Et Statistiques Paris Cité Bobigny France; ^5^ Department of Diabetology, Assistance Publique‐Hôpitaux de Paris (AP‐HP) Pitié‐Salpêtrière University, Nutrition and Obesities: Systemic Approaches, NutriOmics, Research Unit, Sorbonne Université Paris France; ^6^ Centre d'Investigation Clinique 1423 INSERM, Hôpital de la vision des 15‐20 Paris France; ^7^ Service de Diabètes et Endocrinologie APHP INSERM U1138, Hôpital Lariboisière Paris France; ^8^ Service d'Endocrinologie‐Diabétologie‐Nutrition Centre Hospitalier Universitaire de Rennes, Hôpital Sud Rennes France; ^9^ Institut du thorax INSERM, CNRS, UNIV Nantes, CHU Nantes Nantes France; ^10^ Department of Diabetology, Metabolic Diseases and Nutrition CHU Toulouse, University of Toulouse Toulouse France; ^11^ Service d’endocrinologie, diabète et nutrition CHRU Strasbourg Strasbourg France; ^12^ IPE Paris France; ^13^ Centre d'Ophtalmologie Paris Breteuil Paris France; ^14^ INSERM U1151, CNRS UMR 8253, IMMEDIAB Laboratory Institut Necker‐Enfants Malades Paris France; ^15^ Department of Diabetology Endocrinology AP‐HP, Bichat Hospital Paris France; ^16^ Department of Endocrinology, Diabetes, Nutrition Montpellier University Hospital Montpellier France; ^17^ Institute of Functional Genomics University of Montpellier, CNRS, Inserm Montpellier France; ^18^ Endocrinology and Diabetes Department CHU Côte de Nacre Caen France; ^19^ Diabetology Department Cochin Hospital, AP‐HP Paris France; ^20^ Endocrinology, Diabetology, Metabolism and Nutrition Department Lille University Hospital Lille France; ^21^ Service d'Endocrinologie, Centre de Référence des Maladies Rares de l'Insulino‐Sécrétion et de l'Insulino‐Sensibilité (PRISIS) Hôpital Saint‐Antoine, Assistance Publique‐Hôpitaux de Paris Paris France; ^22^ Centre de Recherche Saint‐Antoine, Institut de Cardio‐Métabolisme et Nutrition (ICAN), UMRS 938 Sorbonne Université, Inserm Paris France; ^23^ Department of Endocrinology‐Diabetology, Inserm LNC UMR1231 University of Burgundy Dijon France; ^24^ Service de Diabètes et Endocrinologie Hôpital Lariboisière, APHP Paris France; ^25^ Équipe Immunité et métabolisme du diabète Université Paris Cité, INSERM UMR‐S1151, CNRS UMR‐S8253, Institut Necker Enfants Malades Paris France

**Keywords:** ADDQoL, DCSI, diabetes‐related complications, EQ‐5D‐5L, health‐related quality of life, type 1 diabetes

## Abstract

**Aims:**

Health‐related quality of life (HRQoL) assessment is increasingly integrated into type 1 diabetes (T1D) monitoring to promote a holistic approach. To investigate HRQoL in adults with T1D and to assess the impact of the severity of complications on HRQoL.

**Materials and Methods:**

This is a cross‐sectional analysis of baseline characteristics of adults living with T1D included in Société Francophone du Diabète – Cohorte Diabète de Type 1 (SFDT1), a French longitudinal cohort study. HRQoL was assessed using generic (EuroQol 5‐Dimensions 5‐Level questionnaire [EQ‐5D‐5L]) and diabetes‐specific (Audit of Diabetes‐Dependent Quality of Life) instruments. The severity of diabetes complications was measured using an adapted Diabetes Complication Score Index (DCSI) ranging from 0 to 14. We used multiple imputations to deal with missing data.

**Results:**

We included 1892 adults, 48% women, with a median (interquartile range [IQR]) age of 38 (28; 51) years. The mean overall EQ‐5D‐5L HRQoL score was 71.1 ± 17.7 (maximum 100), with the following number of participants negatively impacted for each domain: 271 (14%) for mobility, 94 (5%) for self‐care, 378 (19%) for usual activities, 853 (45%) for pain/discomfort and 983 (52%) for anxiety/depression. The median (IQR) DCSI was 1 (0; 2). In multivariable models, a one‐step increase in DCSI was associated with a 1.5% decrease in overall EQ‐5D‐5L HRQoL. DCSI was also inversely associated with all domains of the generic scale except anxiety/depression and 17 domains of the diabetes‐specific scale.

**Conclusions:**

We observed an inverse association between the severity of complications and overall HRQoL and most of its dimensions. Our results highlight the need to reinforce the prevention of complications to improve the overall well‐being of people with T1D.

## 1 INTRODUCTION

Type 1 diabetes (T1D) is a chronic disease due to β‐cell autoimmune destruction in the pancreatic islets of Langerhans and with the consequence of having insulin deficiency.^1^ Treatment is burdensome, requiring multiple daily insulin administrations, considering various factors affecting insulin needs. Additionally, there are risks of acute events such as diabetic ketoacidosis and hypoglycaemic coma, as well as chronic complications, including microangiopathy and cardiovascular disease.^1^ This burden related to the condition and its treatment can, as in many chronic conditions, impact health‐related quality of life (HRQoL).^2^ Several studies have shown a decrease in HRQoL among people living with type 1 diabetes (pwT1D).^3^, ^4^ While for many years, the main focus was on assessing glycaemic control and complications screening, the importance of a holistic, person‐centred approach is now well recognised.^5^ This approach includes evaluating the HRQoL of people living with chronic pathology.

The World Health Organization defines quality of life as ‘an individual's perception of their position in life, in the context of the culture and value systems in which they live, and concerning their goals, expectations, standards, and concerns.’^6^ Various tools exist to assess HRQoL. One tool involves asking the patients about their HRQoL with open‐ended questions. Others involve standardised questionnaires grouped under person‐reported outcome measures (PROMs).^7^ The patients complete PROMs directly and describe their experiences and the impact of the condition and treatments on their HRQoL. PROMs can be generic, addressing the entire population or specific to a particular condition.

Several studies have assessed HRQoL in people living with T1D using generic or specific questionnaires. Some of these studies have examined factors associated with HRQoL, such as sex, glycaemic control or duration of diabetes.^3^, ^4^, ^8^, ^9^ An association with diabetes complications has also been studied by analysing the presence or absence of one or more complications or by focusing on a specific complication. However, patients may have several complications of varying severity, and the pathophysiological mechanisms of chronic complications are interlinked.

Our study's objective was to investigate HRQoL among adults living with T1D and assess the impact of severity and number of complications on that HRQoL.

## 2 METHODS

### 2.1 Patients

Société Francophone du Diabète – Cohorte Diabète de Type 1 (SFDT1) is a cohort study of people living with T1D in France. The study was initiated by the French‐speaking Diabetes Society (Société Francophone du Diabète [SFD]), and the acronym SFDT1 is a combination of SFD and DT1 (the French abbreviation for T1D). This study is a cross‐sectional analysis involving patients enrolled in 61 centres between June 2020 and October 2023 (ClinicalTrials.gov identifier: NCT04657783).^10^ Inclusion criteria were age 18 years or older and completion of HRQoL questionnaires by the patients. The National Committee of Ethics in France approved the study (Comité de protection des personnes Ouest V‐RENNES, N° ID‐RCB: 2019‐A01681‐56) in December 2019.

### 2.2 Data collected

HRQoL was assessed using two self‐reported questionnaires: a generic EuroQol 5‐Dimensions 5‐Level questionnaire (EQ‐5D‐5L) and a diabetes‐specific HRQoL questionnaire, evaluated with the third version of the Audit of Diabetes‐Dependent Quality of Life (ADDQoL).

The EQ‐5D‐5L questionnaire includes a visual analogue scale (EQ5D‐VAS) ranging from zero (worst imaginable health state) to 100 (best imaginable health state) and five items assessing five dimensions: mobility, self‐care, usual activities, pain and discomfort, anxiety and depression.^11^ Each item is rated on five levels: one corresponding to the absence of symptoms or problems for that dimension and five to complete inability or extremely present symptoms.

The ADDQoL questionnaire is widely used and validated. The first part of the ADDQoL consists of two scales that assess global HRQoL and the impact of diabetes on HRQoL.^12^, ^13^ The second part includes 19 domains evaluating subdomains of HRQoL: leisure activities, working life, local and long‐distance journeys, holidays, physical health, family life, friendship and social life, close personal relationships, sex life, physical appearance, self‐confidence, motivation, people's reactions, feelings about the future, financial situation, living conditions, dependence on others, freedom to eat and freedom to drink. Each domain has two questions, one evaluating the impact (rated from −3 to +1) and the other evaluating the importance the person attributes to that domain (rated from 0 to +3). These two questions allow the calculation of a weighted score for each domain, ranging from −9 (poor HRQoL) to +3 (good HRQoL). The average score of the 19 domains is calculated: ADDQoL average‐weighted impact score (awADDQoL).

To assess the burden of diabetes complications, we used the Diabetes Complication Score Index (DCSI). Young et al. initially created the DCSI based on data from the International Classification of Diseases (ICD)‐9 coding and inspired by the work of Rosenzweig et al.^14^, ^15^ With the advent of ICD‐10, Glasheen et al. modified the DCSI.^16^ The DCSI consists of seven subsections corresponding to complications: retinopathy, nephropathy, neuropathy, cerebrovascular events, cardiovascular complications, peripheral vascular disease and metabolic complications. Each is scored from 0 to 2, depending on the severity level. In the original score, neuropathy could only take two levels, 0 or 1; we chose to add Level 2, which seemed to correspond more to clinical severity and the high and very high‐risk levels proposed by Rosenzweig et al.^15^ We used a similar approach for retinopathy, with Level 1 corresponding to low or moderate risk and Level 2 to high or very high risk of cecity. Additionally, concerning nephropathy, we used albuminuria and estimated glomerular filtration rate (eGFR) to assess risk based on American Diabetes Association (ADA) and Kidney Diseases Improving Global Outcomes (KDIGO) recommendations.^17^, ^18^ Table S1 shows the adapted score in detail.

Deprivation status associated with indicators of access to health care was evaluated using the Evaluation of Precarity and Inequalities in Health Examination Centers (Evaluation de la Précarité et des Inégalités de santé dans les Centres d'Examens de Santé [EPICES]) score.^19^ Physical activity was assessed using the International Physical Activity Questionnaire (IPAQ)—short version,^20^ which allows calculating the weekly metabolic equivalent of a task (MET minutes per week).

Other collected data included age, sex, body mass index (BMI, kg/m^2^), diabetes duration (years), insulin treatment (multiple daily injections, pump, hypoglycaemia minimiser, automated insulin delivery systems), insulin dose/weight ratio (IU/kg), Glycated hemoglobin (HbA1c) (%), diabetic retinopathy (DR) and its severity, nephropathy as defined by ADA guideline, neuropathy defined by clinical Michigan score (>2),^1^, ^21^ cardiovascular disease (angina pectoris, acute coronary syndrome, coronary angioplasty or bypass, stroke, transient ischemic attack, carotid artery surgery, lower limbs angioplasty, surgery or amputation, history of arrhythmia and hospitalisation for heart failure), tobacco (current or former smoker), hypertension treatment, statin use, antidepressant treatment use, analgesic medication use, marital status and if participants had children.

### 2.3 Statistical methods

Depending on the normal distribution, continuous variables were described using the mean and standard deviation (SD) or the median and interquartile range (IQR), and categorical variables were described using counts and percentages.

For the first part of the analyses, the population was divided into four groups based on quartiles of the generic HRQoL scales (EQ5D‐VAS). Then, for the second part of the analyses, the population was divided into four groups based on quartiles of specific awADDQoL.

The association between DCSI and HRQoL was evaluated using linear regression. The data were adjusted for three models:

1. Model 1 (M1): age, sex and social deprivation score
2. Model 2 (M2): Model 1 plus HbA1c, method of insulin administration and duration of diabetes
3. Model 3 (M3): Model 2 plus smoking, BMI and physical activity.

The ADDQoL score (global HRQoL) ranges from −3 to +3 on a 7‐point scale. We provided percentages of change by dividing the coefficient by 7 × 100. The ADDQoL score to evaluate the impact of diabetes on HRQoL ranges from −3 to +1 on a 4‐point scale. We provided percentages of change by dividing the coefficient by 4 × 100.

Missing values were imputed with multiple imputations using the chained equations approach (Table S2). The estimates were pooled across 40 imputed datasets, and the confidence intervals were calculated according to Rubin rules.

All statistical analyses were performed using R software version 4.3.1.^22^ We used the following R packages: ‘tidyverse,’ ‘gtsummary,’ ‘mice,’ and ‘forest plot.’^23^, ^24^, ^25^, ^26^

## 3 RESULTS

### 3.1 Generic health‐related quality of life scale: EQ‐5D‐5L

#### 3.1.1 Population description

The initial sample included 1964 adult participants; as the EQ‐5D‐5L questionnaire was missing for 72 participants, the final analysis included 1892 participants. The median age of participants was 38 years (IQR: 28; 51), and 52% were male. The median duration of diabetes was 21 years (IQR: 12; 32); 1747 (92%) patients used continuous glucose monitoring (CGM), mainly flash monitoring and 56% used insulin pumps. The median DCSI was 1 point (IQR: 0; 2), with 44% of participants having a DCSI of 0, 21% of 1, 15% of 2 and 20% of more than 2. DR is reported in 687 participants (36%), nephropathy in 378 participants (20%), neuropathy in 335 participants (18%) and cardiovascular disease in 142 participants (7.5%) (Table 1).

**TABLE 1:** Patients' characteristics according to the health‐related quality of life using the EuroQol 5‐Dimensions 5‐Level questionnaire (EQ‐5D‐5L) visual analogue scale.

Characteristic	EQ‐5D visual analogue scale				
	Overall	Group 1: 0–60	Group 2: 61–74	Group 3: 75–80	Group 4: 81–100
	*N* = 1892^a^	*N* = 523^a^	*N* = 427^a^	*N* = 479^a^	*N* = 463^a^
DCSI	1 (0; 2)	1 (0; 3)	1 (0; 2)	1 (0; 2)	0 (0; 2)
Age (years)	38 (28; 51)	41 (29; 53)	35 (28; 48)	38 (28; 51)	37 (28; 49)
Sex					
Female	908 (48%)	299 (57%)	204 (48%)	209 (44%)	196 (42%)
Male	984 (52%)	224 (43%)	223 (52%)	270 (56%)	267 (58%)
BMI (kg/m^2^)	26.0 ± 4.9	26.3 ± 5.6	26.0 ± 4.7	25.9 ± 4.6	25.6 ± 4.5
Diabetes duration (years)	21 (12; 32)	23 (14; 33)	21 (11; 30)	21 (12; 32)	20 (12; 30)
Insulin treatment					
MDI	825 (44%)	236 (45%)	183 (43%)	213 (44%)	193 (42%)
Pump	781 (41%)	214 (41%)	171 (40%)	198 (41%)	197 (43%)
Hypoglycaemia minimiser	79 (4.2%)	19 (3.5%)	22 (5.2%)	14 (2.9%)	24 (5.3%)
AIDS	207 (11%)	54 (10%)	51 (12%)	54 (11%)	48 (10%)
Insulin dose (IU/kg)	0.61 ± 0.28	0.64 ± 0.30	0.62 ± 0.28	0.61 ± 0.27	0.58 ± 0.25
CGM	1747 (92%)	477 (91%)	395 (93%)	447 (93%)	428 (92%)
HbA1c (%)	7.69 ± 1.33	8.00 ± 1.56	7.68 ± 1.21	7.60 ± 1.33	7.43 ± 1.07
Retinopathy	687 (36%)	217 (41%)	163 (38%)	169 (35%)	138 (30%)
DR severity					
No DR	1232 (65%)	314 (60%)	270 (63%)	316 (66%)	332 (72%)
NPDR	510 (27%)	148 (28%)	127 (30%)	127 (26%)	108 (23%)
PDR	150 (7.9%)	61 (12%)	29 (6.9%)	36 (7.5%)	23 (5.1%)
Nephropathy	378 (20%)	134 (26%)	88 (21%)	83 (17%)	72 (16%)
Neuropathy	335 (18%)	139 (27%)	63 (15%)	67 (14%)	67 (15%)
Cardiovascular disease	142 (7.5%)	61 (12%)	27 (6.4%)	34 (7.1%)	19 (4.2%)
Current and former tobacco use	832 (44%)	257 (49%)	189 (44%)	221 (46%)	164 (35%)
Hypertension	370 (20%)	142 (27%)	81 (19%)	81 (17%)	67 (14%)
Statin use	508 (27%)	164 (31%)	106 (25%)	132 (28%)	105 (23%)
Antidepressant treatment use	120 (6.3%)	67 (13%)	26 (6.0%)	15 (3.2%)	12 (2.6%)
Analgesic medication use	74 (3.9%)	50 (9.6%)	12 (2.9%)	8 (1.7%)	4 (0.9%)
Marital status					
Couple	1189 (63%)	303 (58%)	264 (62%)	313 (65%)	310 (67%)
Single	703 (37%)	220 (42%)	163 (38%)	166 (35%)	153 (33%)
Have children	935 (49%)	271 (52%)	189 (44%)	243 (51%)	232 (50%)
Social deprivation score	17 (8; 30)	24 (14; 40)	17 (8; 30)	17 (7; 28)	15 (7; 24)
Physical activity (MET)	1866 (918; 3279)	1627 (708; 3024)	1832 (960; 3276)	1936 (975; 3339)	2034 (1004; 3459)

*Note*: Four health‐related quality of life (HRQoL) groups were defined according to the quartiles of the EQ5D‐VAS scale, with Group 1 corresponding to patients reporting a lower HRQoL and Group 4 to those reporting a better HRQoL.

Abbreviations: AIDS, automated insulin delivery systems; BMI, body mass index; CGM, continuous glucose monitoring; DCSI, Diabetes Complication Score Index; DR, diabetic retinopathy; EQ‐5D, EuroQoL 5‐Dimensions; MET, metabolic equivalent of task; MDI, multiple daily injections; NPDR, non‐proliferative diabetic retinopathy; PDR, proliferative diabetic retinopathy.

^a^ Median (interquartile range), mean ± standard deviation or *n* (%) depending on the conditions of application.

The mean overall HRQoL assessed by EQ5D‐VAS was 71.1 ± 17.7 (/100), and the median was 74 (IQR: 60; 80). Table S4 describes the overall HRQoL according to age groups, with the data categorised into 10‐year age brackets.

Among participants, 271 (14%) reported decreased HRQoL in mobility, 94 (5%) in self‐care, 378 (19%) in usual activities, 853 (45%) in pain and discomfort and 983 (52%) in anxiety and depression (Table S3). The population was then divided into three age groups: young (18–24 years), middle‐aged (25–64 years) and elderly (65 years and over). The young group reported 4% of mobility impairment, 1.3% of self‐care impairment, 13% of usual activities impairment, 30% of pain and discomfort and 49% in anxiety and depression. The middle‐aged group reported 15% of mobility impairment, 5.3% of self‐care impairment, 21% of usual activities impairment, 47% of pain or discomfort and 54% in anxiety and depression. The elderly group reported 41% of mobility impairment, 10% of self‐care impairment, 22% of usual activities impairment, 64% of pain or discomfort and 34% in anxiety and depression.

#### 3.1.2 Association between health‐related quality of life and severity of diabetes complications

**FIGURE 1:**
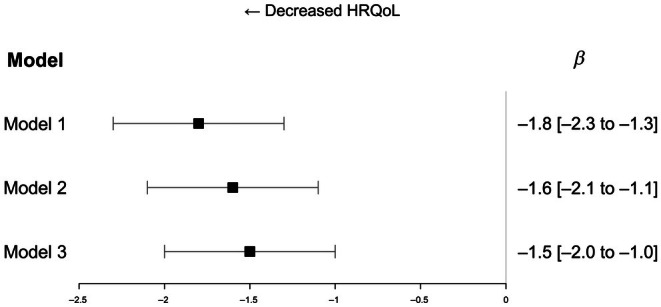
Association between global health‐related quality of life (EuroQoL 5‐Dimensions visual analogue scale) and severity of diabetes complications (Diabetes Complication Score Index [DCSI]). Model 1: DCSI adjusted for age, gender and social deprivation score; Model 2: Model 1 and diabetes duration, HbA1c and insulin administration mode; Model 3: Model 2 and body mass index, smoker and physical activity. Confidence intervals were calculated according to Rubin rules. HRQoL, health‐related quality of life.

Table 1 describes the population's characteristics based on the overall HRQoL (EQ5D‐VAS). After adjustment for age, gender, social deprivation score, diabetes duration, HbA1c, insulin administration mode, BMI, smoking status and physical activity, an association was observed between EQ5D‐VAS and severity of complications (DCSI), regardless of the model used (Figure 1). Each increase in the DCSI score was associated with a 1.5% decrease in the global HRQoL measured by the EQ‐5D‐5L questionnaire (*β*: −1.5, 95% CI −2.0 to −1.0, *p* < 0.001). A significant association was found between decreased HRQoL and a higher social deprivation score, being female, and a higher HbA1c level (Table S5).

**FIGURE 2:**
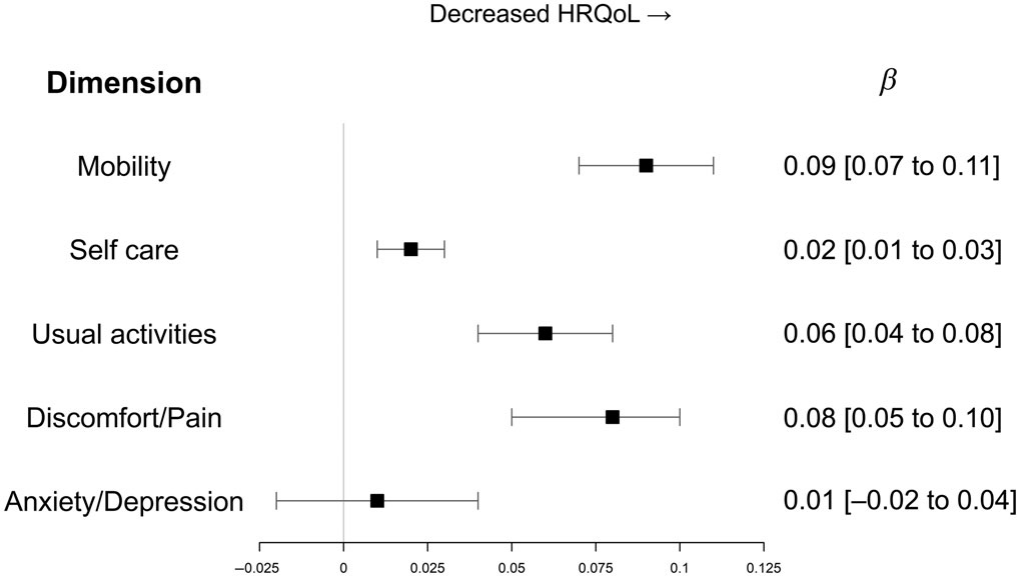
Association between health‐related quality of life dimensions (5‐Dimensions visual analogue scale) and severity of diabetes complications (Diabetes Complication Score Index score [DCSI]). Model 3: DCSI adjusted for age, gender, social deprivation score, diabetes duration, HbA1c, insulin administration mode, body mass index, tobacco and physical activity. Confidence intervals were calculated according to Rubin rules. HRQoL, health‐related quality of life.

Four dimensions of HRQoL showed an inverse association with the severity of complications, but one (anxiety and depression) did not (Figure 2).

### 3.2 Specific scale of health‐related quality of life in people living with diabetes: ADDQoL

#### 3.2.1 Population description

In this second part of the analysis, we included 1375 patients out of the initial sample of 1964 participants who filled out the ADDQoL questionnaire. The median age of participants was 39 years (IQR: 29; 51), and 50% were male. The median duration of diabetes was 22 years (IQR: 13; 32); 1287 (94%) of participants used CGM and 830 (60%) used insulin pumps. The median DCSI was 1 point (IQR: 0; 2), with 45% of participants having a DCSI of 0, 23% of 1, 15% of 2 and 17% of more than 2. DR is reported in 489 participants (36%), nephropathy in 272 participants (20%), neuropathy in 228 participants (17%) and cardiovascular disease in 100 participants (7.3%) (Table 2).

**TABLE 2:** Characteristics of the study sample according to health‐related quality of life using the Audit of Diabetes‐Dependent Quality of Life (ADDQoL)‐specific scale.

Characteristic	ADDQoL average‐weighted impact score				
	Overall	Group A: −9 to −3.6	Group B: −3.5 to −2.3	Group C: −2.2 to −1.3	Group D: −1.2 to 3
	*N* = 1375^a^	*N* = 348^a^	*N* = 340^a^	*N* = 352^a^	*N* = 335^a^
DCSI	1 (0; 2)	1 (0; 3)	1 (0; 2)	1 (0; 2)	1 (0; 2)
Age (years)	39 (29; 51)	38 (30; 50)	39 (29; 50)	38 (28; 50)	41 (30; 52)
Sex					
Female	687 (50%)	203 (58%)	180 (53%)	159 (45%)	145 (43%)
Male	688 (50%)	145 (42%)	160 (47%)	193 (55%)	190 (57%)
BMI (kg/m^2^)	26.1 ± 4.9	26.4 ± 5.1	25.9 ± 4.8	26.1 ± 4.9	26.1 ± 4.9
Diabetes duration (years)	22 (13; 32)	22 (14; 32)	22 (12; 32)	21 (11; 32)	23 (15; 34)
Insulin treatment					
MDI	546 (40%)	134 (39%)	125 (37%)	150 (43%)	137 (41%)
Pump	608 (44%)	156 (45%)	164 (48%)	149 (42%)	139 (42%)
Hypoglycaemia minimiser	63 (4.6%)	16 (4.7%)	17 (5.1%)	15 (4.3%)	14 (4.2%)
AIDS	159 (12%)	41 (12%)	34 (10%)	38 (11%)	45 (13%)
Insulin dose (IU/kg)	0.61 ± 0.28	0.61 ± 0.27	0.60 ± 0.28	0.60 ± 0.27	0.62 ± 0.30
CGM	1287 (94%)	320 (92%)	317 (93%)	332 (94%)	318 (95%)
HbA1c (%)	7.63 ± 1.29	7.82 ± 1.39	7.58 ± 1.24	7.55 ± 1.32	7.54 ± 1.16
Retinopathy	489 (36%)	141 (41%)	128 (38%)	116 (33%)	104 (31%)
DR severity					
No DR	906 (66%)	210 (60%)	219 (64%)	242 (69%)	235 (70%)
NPDR	377 (27%)	104 (30%)	98 (29%)	94 (27%)	81 (24%)
PDR	93 (6.7%)	35 (10%)	23 (6.9%)	16 (4.4%)	19 (5.7%)
Nephropathy	272 (20%)	86 (25%)	58 (17%)	67 (19%)	61 (18%)
Neuropathy	228 (17%)	83 (24%)	56 (17%)	45 (13%)	44 (13%)
Cardiovascular disease	100 (7.3%)	29 (8.3%)	23 (6.8%)	25 (7.1%)	23 (6.8%)
Current and former tobacco use	605 (44%)	159 (46%)	148 (44%)	167 (47%)	131 (39%)
Hypertension	264 (19%)	70 (20%)	59 (17%)	64 (18%)	71 (21%)
Statin use	383 (28%)	98 (28%)	99 (29%)	94 (27%)	93 (28%)
Antidepressant treatment	94 (6.8%)	45 (13%)	23 (6.8%)	18 (5.2%)	8 (2.4%)
Analgesic medication	50 (3.6%)	19 (5.5%)	13 (3.8%)	8 (2.3%)	9 (2.7%)
Marital status					
Couple	898 (65%)	216 (62%)	218 (64%)	239 (68%)	225 (67%)
Single	477 (35%)	132 (38%)	122 (36%)	113 (32%)	110 (33%)
Have children	717 (52%)	183 (53%)	184 (54%)	168 (48%)	182 (54%)
Social deprivation score	17 (7; 28)	22 (12; 37)	17 (7; 30)	15 (7; 25)	15 (7; 21)
Physical activity (MET)	1902 (950; 3348)	2076 (993; 3690)	1866 (924; 3400)	1836 (975; 3279)	1914 (918; 3173)

*Note*: Four health‐related quality of life (HRQoL) groups were defined according to the quartiles of the weighted mean ADDQoL score, with Group A corresponding to patients reporting a lower HRQoL and Group DCV has spoken at conferences organised by the following pharmaceutical companies: Abbott, Advanz Pharma, Amryt, AstraZeneca, Eli Lilly and Company, Novartis, Novo Nordisk and Sanofi. to those reporting a better HRQoL.

Abbreviations: AIDS, Automated insulin delivery systems; BMI, body mass index; CGM, continuous glucose monitoring; DCSI, Diabetes Complication Score Index; DR, diabetic retinopathy; EQ‐5D, EuroQoL 5‐Dimensions; MET, metabolic equivalent of task; MDI, multiple daily injections; NPDR, non‐proliferative diabetic retinopathy; PDR, proliferative diabetic retinopathy.

^a^ Median (interquartile range), mean ± standard deviation or *n* (%) depending on the conditions of application.

The mean global HRQoL was 0.99 ± 0.96 on a scale ranging from −3 to +3: 45 (3.3%) of the participants described it as excellent, 341 (25%) as very good, 648 (47%) as good, 261 (19%) as neither good nor bad, 58 (4.2%) as bad, 16 (1.2%) as very bad and 6 (0.4%) as extremely bad. If they had not had diabetes, 323 (23%) of participants estimated that their quality of life would be very much better, 512 (37%) much better, 417 (30%) a little better, 103 (7.5%) the same and 20 (1.5%) worse, with an average of −1.74 ± 0.95 on a scale from −3 (very much better) to 1 (worse).

Regarding specific domains of HRQoL, mean weighted scores varied from −3.77 ± 3.17 for freedom to eat to −0.97 ± 1.99 for people's reactions (Table S6 and Figure S1). The average weighted score (awADDQoL) was −2.60 ± 1.74, and the median was −2.32 (IQR: −3.58; −1.26).

#### 3.2.2 Association between diabetes‐specific quality of life and severity of diabetes complications

After controlling for age, gender, social deprivation score, diabetes duration, HbA1c, insulin administration mode, BMI, smoking status and physical activity, each increase in the DCSI score was associated with a 0.9% decrease in the global HRQoL measured by the ADDQoL questionnaire (*β*: −0.06, 95% CI −0.10 to −0.03, *p* < 0.001). We also examined the question, ‘If I did not have diabetes, my quality of life would be … very much better/much better/a little better/the same/worse.’ We found that each increase in one point of the DCSI score was associated with a 2% decrease in the score (*β*: −0.08, 95% CI −0.12 to −0.05, *p* < 0.001). Thus, an increase in DCSI is associated with a greater impact of having diabetes on global HRQoL.

**FIGURE 3:**
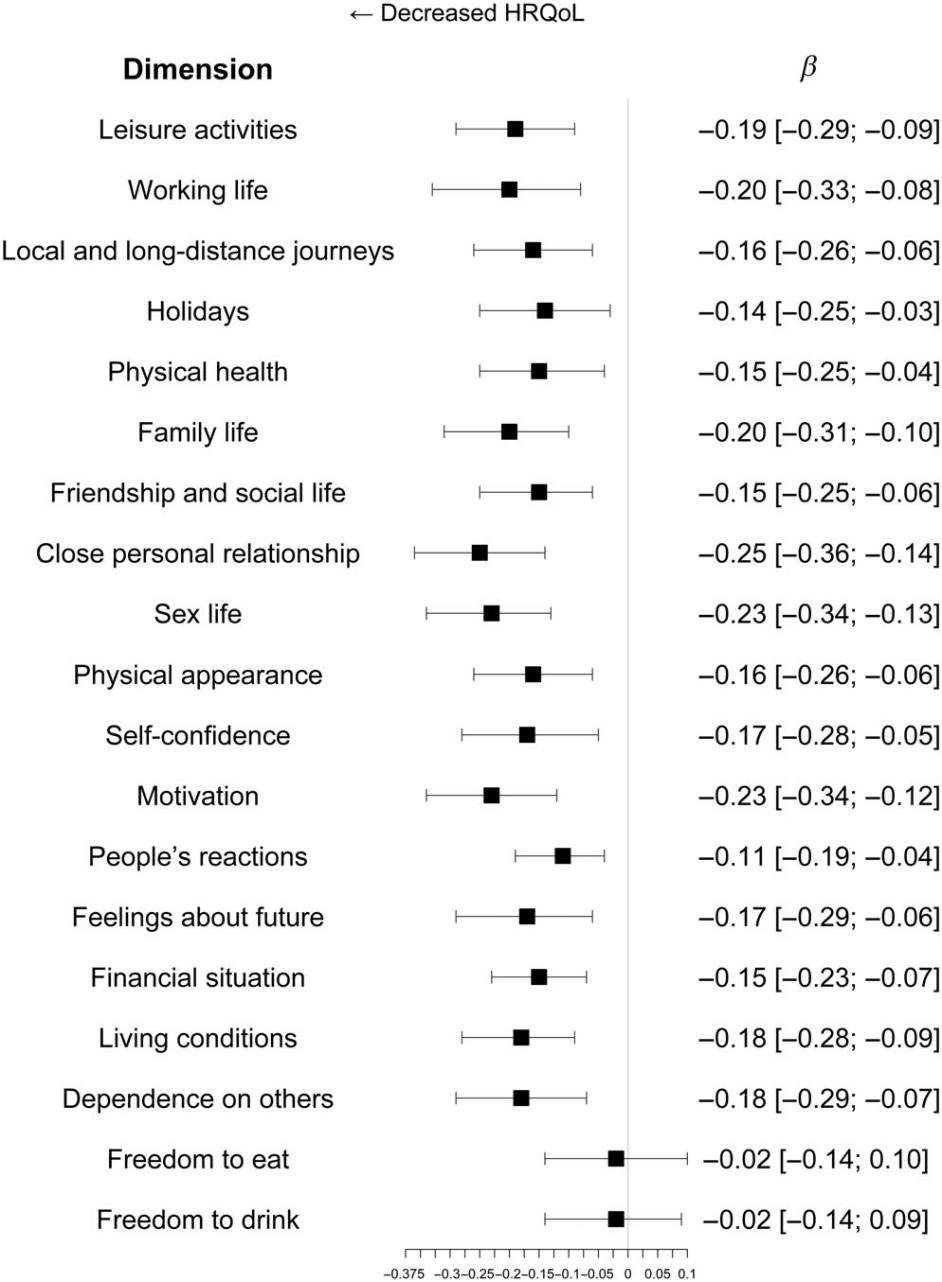
Association between health‐related quality of life dimensions (Audit of Diabetes‐Dependent Quality of Life) and severity of diabetes complications (Diabetes Complication Score Index [DCSI] score). Model 3: DCSI adjusted for age, gender, social deprivation score, diabetes duration, HbA1c, insulin administration mode, body mass index, tobacco and physical activity. Confidence intervals were calculated according to Rubin rules. HRQoL, health‐related quality of life.

The mean weighted score of the various specific domains of HRQoL was inversely associated with the severity of complications (*β*: −0.16, confidence interval from −0.23 to −0.10, *p* < 0.001). Finally, the various specific domains, excluding freedom to eat and freedom to drink, were also inversely associated with DCSI (Figure 3).

## 4 DISCUSSION

This work shows an inverse association between overall HRQoL and the number and severity of complications in a large population of pwT1D. This association is also present in various aspects of HRQoL, whether general or specific to diabetes problems.

Other studies have assessed HRQoL in pwT1D using generic questionnaires and compared it to the general population. In the Netherlands, in 2003, a first study found a mean EQ5D‐VAS score of 80.8/100 in a population of 281 pwT1D of similar age and sex as in our research.^27^ The score level was lower than that of the general population. The control group came from a study on the United Kingdom general population. More recently, in Sweden and Denmark, the decline in HRQoL was confirmed in two studies, including 839 and 2415 pwT1D who were matched to a control group selected from the general population.^3^, ^4^ Although our study did not include a control group, we can compare the results to those of a recent 2018 survey of the French general population, in which the average EQ5D‐VAS score was 73.4 compared to 71 in the present study.^28^ However, the difference appears more pronounced for the youngest participants. Thus, in the 18–24 age category, the SFDT1 participants had an average EQ5D‐VAS score of 72 compared to 78 in the general population. The SFDT1 population frequently reported impairment in the domain of anxiety/depression and discomfort/pain. This is also the case in the general population. The impairment in the domain of anxiety/depression was particularly marked in women in both studies. These studies and ours clearly show the impact of T1D on HRQoL, particularly in the youngest. To improve their HRQoL, exploring the factors contributing to its alteration is essential.

To study the link between diabetes complications and HRQoL, we used a severity score rather than taking into account complications individually. On the one hand, the leading chronic complications are linked to vascular damage to different organs, whose pathophysiological mechanisms are intertwined.^29^ On the other hand, the same patient can present several complications simultaneously, whether acute or chronic. Thus, integrating the different complications in the same score and considering precariousness seemed more appropriate to represent the patient globally, using a holistic approach. However, we acknowledge that this approach has limitations. The DCSI, while providing a comprehensive assessment of the overall burden of complications, may not fully capture the differential impact of specific complications (e.g., neuropathy vs. retinopathy) on HRQoL. For example, a stroke probably has a greater impact on mobility and autonomy, whereas neuropathy is more likely to be associated with a decline in HRQoL in the pain and discomfort domain. The DCSI is a score already described in many studies, mainly for determining morbidity and mortality or stratifying health costs.^30^, ^31^, ^32^, ^33^ It was built from ICD‐9 and then ICD‐10 data, so we had to adapt it to integrate clinical and biological data. This made it even more specific, particularly concerning nephropathy.

Some studies have examined the link between HRQoL and diabetes complications. The studies that include the most significant number of patients are those looking at diabetes overall or type 2 diabetes. Fu et al. report, in particular, a drop of 9.2 points in EQ5D‐VAS in the presence of macrovascular complications in a population of around 3000 people.^34^ Studies focusing on T1D have a smaller sample size, ranging from 108 to 520 patients.^8^, ^27^, ^35^, ^36^ Two of them found an association between cardiovascular diseases and HRQoL, one with proliferative DR, one with various microangiopathic complications, one with microangiopathic complications, and finally, one with lower limb amputations. The main questionnaires used are the Short Form‐36 (SF‐36) and the EQ‐5D.

This work highlights an association between the decline in overall HRQoL and the severity of complications. This is rare considering only pwT1D. Morgan et al. reported, in 4502 patients, a decrease in the EQ‐5D index in patients with diabetes by categories of complications: none, single one and several ones.^37^ Mujica‐Mota et al. showed, in the general population, including more than a million people and using self‐reported questionnaires, a decrease in HRQoL in the case of multimorbidities.^38^ The only study we found concerning T1D included 111 patients and found an association with the number of complications of diabetes.^39^

Beyond the overall HRQoL, we find the same type of link between the severity of complications and the various dimensions of HRQoL, whether generic or specific. This highlights the impact of complications on different aspects of the lives of pwT1D and reinforces the importance of preventing complications to maintain quality of life.

Only three dimensions appeared not to be involved: anxiety/depression, freedom to drink and freedom to eat. The absence of an association between anxiety/depression and HRQoL was unexpected. This result likely reflects the fact that the EQ‐5D‐5L captures the participant's current emotional state rather than a formal psychiatric diagnosis. Consistent with the general population study by Gautier et al., women in our sample were more likely to report anxiety or depression.^28^ Furthermore, in contrast to the other domains, there is no increase in complaints with age. Unlike other HRQoL domains, we observed no increase in anxiety/depression complaints with age, suggesting that these symptoms may be more closely linked to the psychosocial burden of living with diabetes itself—or to external difficulties related to gender or life stage—rather than to the progression of complications. This interpretation is further supported by the behaviour of the other two diabetes‐specific dimensions—freedom to drink and freedom to eat—which are directly influenced by the condition and its treatment. The lack of association between these dimensions and complication severity is logical, as they are primarily affected by dietary restrictions and treatment regimens, as demonstrated by The Dose Adjustment for Normal Eating (DAFNE) study's findings on improved quality of life following carbohydrate counting training.^40^ The absence of a clear link between complication severity and anxiety/depression may also highlight the limitations of the EQ‐5D‐5L in capturing the nuanced mental health impacts of diabetes.^41^ Mental well‐being in this population is influenced by a complex interplay of factors, including self‐management challenges, psychosocial stressors and individual coping mechanisms, which may overshadow the direct effect of physical complications.^42^ Future studies using diabetes‐specific mental health instruments could provide deeper insights into this relationship.

### 4.1 Strengths and limitations

One strength of this study is the sample size. Indeed, the number of studies evaluating HRQoL in adult pwT1D remains limited. Another strength is that the multicentre design also makes it possible to avoid having a hyper‐selected population, thus limiting bias while facilitating the extrapolation of the results to other pwT1D. In addition, we find similar results regardless of the model tested, each including different clinical parameters associated with HRQoL. While our findings provide valuable insights, they are derived from a French cohort with specific demographic and healthcare system characteristics, which may limit their generalisability to other populations and settings. Furthermore, it should be noted that these are self‐reported outcomes, and therefore, they may suffer from response bias. They should not be interpreted as clinical symptoms or pathologies as such, particularly concerning the anxiety/depression dimension, which cannot be taken in the psychiatric sense of the term. Furthermore, we used the DCSI, which is usually not used in studies including only pwT1D. Finally, the study's cross‐sectional design does not allow us to confirm a causal link, which a longitudinal analysis and another cohort must confirm.

## 5 CONCLUSION

This study shows an inverse association between HRQoL and the severity of diabetes complications, whether it is the overall HRQoL or the various generic or specific dimensions, except for anxiety/depression, freedom to drink and freedom to eat. Thus, the increase in the severity level of complications leads to a 1.5% decrease in overall HRQoL, showing the burden of diabetes complications for pwT1D. To illustrate this result, consider two pwT1D who have similar characteristics apart from one having no complications and the other having mild non‐proliferative DR, peripheral neuropathy, and a history of myocardial infarction. The HRQoL would be 6% lower for the second person. This highlights how preventing complications can contribute to the overall well‐being of people living with diabetes.

## AUTHOR CONTRIBUTIONS

SB, EC, CA, SF, J‐FG, PV, SH, HH, LK, EL‐S, PM, LP, ER, YR, AS, AV, CV, BV, J‐PR and GF contributed to protocol development and study design. GF conceptualised the study. J‐PR and EC are the co‐principal investigators. SB, GAA, EC, CA, J‐FG, PV, SH, HH, LK, EL‐S, PM, LP, ER, YR, AS, AV, CV, BV and J‐PR participated in patients' inclusion. GAA performed data curation. GF, GAA, EC, J‐PR, PM and SB contributed to the Methodology. SB performed the formal analysis. GF and GAA performed supervision of analyses. SB prepared the figures and wrote the original draft. SB, GAA, EC, CA, SF, J‐FG, PV, SH, HH, LK, EL‐S, PM, LP, ER, YR, AS, AV, CV, BV, J‐PR and GF reviewed and edited the draft. SB, GAA, GF, J‐PR and EC had full access to the data in the study, verified the data and had full responsibility for the decision to submit and publish.

## FUNDING INFORMATION

This work was made possible thanks to the institutional support from the Foundation Francophone pour la Recherche sur le Diabète (FFRD), the Société Francophone du Diabète (SFD) and the Luxembourg Institute of Health, as well as from the following partners: Breakthrough T1D, Aide aux Jeunes Diabétiques (AJD), Fédération Française des Diabétiques, Lilly, Abbott, Air Liquide Healthcare, Novo Nordisk, Sanofi, Insulet, Medtronic, Dexcom, Ypsomed, Lifescan and Sur les Pas de So. The study sponsors/funders were not involved in the study's design; the collection, analysis and interpretation of data, writing the report, and did not impose any restrictions regarding the publication of the report.

## CONFLICT OF INTEREST STATEMENT

Sara Barraud, Gloria A. Aguayo, Chloé Amouyal, Sylvie Feldman, Jean‐François Gautier, Patricia Vaduva, Samy Hadjadj, Hélène Hanaire, Laurence Kessler, Pascale Massin, Eric Renard, Yves Reznik, Agnès Sola, Anne Vambergue, Bruno Vergès and Guy Fagherazzi have nothing to declare. Emmanuel Cosson has received grants or contracts from Abbott, AstraZeneca, Novartis, Novo Nordisk, Roche Diagnostics and Sanofi. Emmanuel Cosson also performed consulting fees for Abbott, AlphaDiab, Bayer, AstraZeneca, DexCom, Lilly, LVL Medical, Medtronic, Novartis, Novo Nordisk, Roche Diabetes and Sanofi. Emmanuel Cosson received honoraria for lectures, presentations, speakers' bureaus, manuscript writing or educational events from Abbott, Amgen, Bayer, AstraZeneca, Boehringer Ingelheim, Lilly, LVL Medical, Medtronic, Novo Nordisk, Roche Diagnostics, Sanofi, Urgo, ViiV Healthcare (GSK) and Roche Diagnostics. Emmanuel Cosson received support for attending meetings and/or travel from AlphaDiab, Asdia, Lilly, Sanofi and Vitalaire. Jean‐Pierre Riveline received payment or honoraria for lectures, presentations, speakers' bureaus, manuscript writing or educational events from Sanofi Aventis, MSD, Eli Lilly, Novo Nordisk, AstraZeneca, Abbott, Dexcom, AlphaDiab and Medtronic, Abbott, Air Liquide Santé International and Sanofi. Louis Potier reports grants, personal fees and non‐financial support from AstraZeneca, Eli Lilly, Novo Nordisk, Sanofi, Boehringer Ingelheim, Bayer and Glooko XT. Camille Vatier is an investigator in the Regeneron REGN4461‐PLD‐20100 and Amryt APL‐22 therapeutic trials. CV has spoken at conferences organised by the following pharmaceutical companies: Abbott, Advanz Pharma, Amryt, AstraZeneca, Eli Lilly and Company, Novartis, Novo Nordisk and Sanofi. Emmanuelle Lecornet‐Sokol reports non‐financial support from Abbott, AstraZeneca, Boehringer Ingelheim, Dexcom, Lilly, Novo Nordisk and Sanofi. All authors declare that there are no other relationships or activities that might bias, or be perceived to bias, their work.

## Supporting information


**Data S1.** Supporting Information.

## Data Availability

Data used for this analysis are available for academic researchers on request submitted to the scientific committee of SFDT1: cohorte.sfdt1@gmail.com. Codes created for this analysis are available on request to the corresponding author: sbarraud@chu‐reims.fr.
