# Supplementary material for: Severity of complications is associated with impaired health‐related quality of life in people with type 1 diabetes
Source: Diabetes Obes Metab. 2025 Dec 2;28(2):1201–12. doi: 10.1111/dom.70306 (PMC12803608; doi:10.1111/dom.70306)

Supplemental

Table S1: Description of Adapted Diabetes Complication Score Index (DCSI)

	DCSI Score original*				Adapted DCSI score with SFDT1 variables
Complications	ICD-9 Diagnosis**	ICD-9 Code**	1	2	Variables
**Retinopathy**	Diabetic ophthalmologic disease	250.5x	•		NA
	Background retinopathy	362.01	•		Diabetic retinopathy other than PDR
	Other retinopathy	362.1	•		idem
	Retinal edema	362.83	•		NA
	CSME	362.53		••***	Diabetic macular edema
	Other retinal disorders	362.81, 362.82	•		NA
	Proliferative retinopathy	362.02		••	Proliferative retinopathy, PRP DR are considered as PDR and severe NPDR
	Retinal detachment	361.xx		••	complicated PDR
	Blindness	369.xx .00-.99		••	History of visual loss / visual acuity / unilateral or bilateral blindness
	Vitreous hemorrhage	379.23		••	complicated PDR or vitrectomy
**Nephropathy**	Diabetic nephropathy	250.4	•		calculated variable with albuminuria and GFR
	Acute glomerulonephritis	580	•		NA
	Nephrotic syndrome	581	•		NA
	Hypertension, nephrosis	581.81	•		NA
	Chronic glomerulonephritis	582	•		NA
	Nephritis/nephropathy	583	•		NA
	Chronic renal failure	585		••	NA
	Renal NOS	586		••	NA

	Renal insufficiency	593.9	••	NA
	Urine protein >30 mg/g of creatinine, or (+) dipstick protein or serum creatinine >1.5 mg/dL		•	microalbuminuria >= 30 mg/g or >=3 mg/mmol or >= 30 mg/24h or >= 20 mg/L (only if other are NA)
	Serum creatinine >2.0 mg/dL		••	NA replaced by GFR <30 mL/min
			•	GFR ≥30 but <59 mL/min3 (CKD Stage 3) (as proposed by Glasheen *et al.*)
			••	GFR (CKD-EPI) < 30 mL/min3 (CKD Stage 4 or more) (as proposed by Glasheen *et al.*)
			••	End-stage renal disease: dialysis or kidney transplantation
**Neuropathy**	Diabetic neuropathy	356.9, 250.6	•	Clinical Michigan score>2
	Amyotrophy	358.1	•	NA
	Cranial nerve palsy	951.0, 951.1, 951.3	•	NA
	Mononeuropathy	354.0-355.9	•	NA
	Charcot's arthropathy	713.5	••	Charcot's arthropathy
	Polyneuropathy	357.2	•	idem diabetic neuropathy
	Neurogenic bladder	596.54	••	Neurogenic bladder
	Autonomic neuropathy	337.0, 337.1	••	Autonomic neuropathy
	Gastroparesis/diarrhea	564.5, 536.3	••	Gastroparesis and diarrhea/constipation
	Orthostatic hypotension	458.0	••	Orthostatic hypotension
**Cerebrovascular**	TIA	435	•	TIA
	Stroke	431, 433, 434, 436	••	Stroke
			••	precerebral arteries surgery (adapted from Glasheen *et al.*: ICD-10 I65.x)
**Cardiovascular**	Atherosclerosis	440.xx	•	NA
	Other IHD	411	•	Acute coronary syndrome: unstable angina pectoris, other
	Angina pectoris	413	•	Stable angina pectoris
	Other chronic IHD	414	•	Coronary angioplasty
	Myocardial infarction	410	••	Myocardial infarction
	Ventricular fibrillation, arrest	427.1, 427.3	••	Cardiac arrest resuscitated
	Atrial fibrillation, arrest	427.4, 427.5	••	Cardiac arrest resuscitated

	Other ASCVD	429.2	•	Arrhythmia
	Old myocardial infarction	412	••	Coronary artery bypass
	Heart failure	428	••	Hospitalized for heart failure
	Atherosclerosis, severe	440.23, 440.24	••	NA
	Aortic aneurysm/dissection	441	••	NA
**Peripheral vascular disease**	Diabetic PVD	250.7	•	No peripheral pulse
	Other aneurysm, LE	442.3	•	NA
	PVD	443.81, 443.9	•	NA
	Foot wound + complication	892.1	•	foot wood (>=1month)
	Claudication, intermittent	443.9	•	Claudication, intermittent
	Embolism/thrombosis (LE)	444.22	••	LE angioplasty / arterial surgery LE / non-traumatic amputation (LE)
	Gangrene	785.4	••	NA
	Gas gangrene	0.40	••	NA
	Ulcer of lower limbs	707.1	••	NA
**Metabolic**	Ketoacidosis	250.1	••	Ketoacidosis
	Hyperosmolar	250.2	••	NA (only type 1 diabetes)
	Other coma	250.3	••	hypoglycemia with coma or convulsion

*Severity index is based on a scale ranging from 0-2 for each complication as follows: 0 = no abnormality, 1 = some abnormality, 2 = severe abnormality ; **ICD-9 classification ; ***As macular edema can lead to cecity, it has been relevel to 2.

• indicates a count of 1 added to DCSI; indicates a count of 2 added to DCSI.

ASCVD: atherosclerotic cardiovascular disease, ICD-9: International Classification of Diseases, Ninth Revision, IHD, ischemic heart disease, LE, lower extremity, NOS : not otherwise specified, PVD, peripheral vascular disease, TIA, transient ischemic attack.

Table S2: Description of missing data

Variable	Missing (n)	Percent (%)
Age (years)	0	0.0
Sex	0	0.0
Diabetic retinopathy	0	0.0
EQ5D-VAS	0	0.0
Self-care	6	0.3
Usual activity	6	0.3
Mobility	7	0.4
Pain, discomfort	10	0.5
Diabetes duration (years)	18	1.0
Current & Former Tobacco use	19	1.0
Hypertension	19	1.0
Cardiovascular history	19	1.0
Stroke	19	1.0
Myocardial infarction	19	1.0
DCSI cerebrovascular event	19	1.0
DCSI cardiovascular complication	19	1.0
Anxiety, depression	26	1.4
Insulin treatment	30	1.6
Other antidiabetic treatment	30	1.6
Statin use	30	1.6
ACE inhibitor use	30	1.6
ARB2 use	30	1.6
Aspirin use	30	1.6
Antidepressant treatment	30	1.6
Analgesic medication	30	1.6
GCM use	31	1.6
DCSI metabolic	31	1.6
Alcohol	33	1.7
Marital status	33	1.7
Living alone	33	1.7
Have children	33	1.7
Other treatment	44	2.3
Number of other treatments	44	2.3
EQ5D5L index value	46	2.4
Risk Stratification System of foot ulceration	54	2.9
History of foot ulceration	54	2.9

**DCSI retinopathy**	66	3.5
**BMI**	77	4.1
**Proliferative diabetic retinopathy**	80	4.2
**Autonomic neuropathy**	93	4.9
**Clinical Michigan score**	161	8.5
**DCSI peripheral vascular disease**	163	8.6
**DCSI neuropathy**	170	9.0
**Family history of cardiovascular disease**	192	10.1
**Social deprivation score (EPICES)**	208	11.0
**estimated GFR (ml/min)**	236	12.5
**LDL cholesterol (g/L)**	285	15.1
**HbA1C (%)**	425	22.5
**Insulin dose (IU/kg)**	511	27.0
**ADDQoL average weighted score**	517	27.3
**DCSI nephropathy**	632	33.4
**Physical activity (MET/week)**	708	37.4

EQ5D-VAS: EuroQol 5-Dimensions visual analogue scale

Table S3: Description of EQ5D5L

Characteristic	N = 1,892
**EQ5D VAS**	
Mean±SD	71±18
Median (IQR)	74 (60, 80)
Range	0, 100
**Mobility**	
1	1,621 (86%)
2	137 (7.2%)
3	88 (4.6%)
4	38 (2.0%)
5	8 (0.4%)
**Self-care**	
1	1,798 (95%)
2	56 (3.0%)
3	26 (1.4%)
4	5 (0.3%)
5	7 (0.4%)
Usual activities	
1	1,524 (81%)
2	227 (12%)
3	104 (5.5%)
4	30 (1.6%)
5	7 (0.4%)
**Pain, discomfort**	
1	1,039 (55%)
2	463 (24%)
3	280 (15%)
4	93 (4.9%)
5	16 (0.9%)
**Anxiety, depression**	
1	909 (48%)
2	527 (28%)
3	328 (17%)
4	93 (4.9%)
5	35 (1.9%)

EQ5D-VAS: EuroQol 5-Dimensions visual analogue scale

Table S4: Overall HRQoL by Age Group – EQ5D5L

	Characteristic	N = 1,892
EQ5D VAS	18-24 years	72.09 (n=308)
	25-34 years	71.67 (n=538)
	35-44 years	72.26 (n=387)
	45-54 years	68.48 (n=353)
	55-64 years	69.65 (n=244)
	65-74 years	72.68 (n=82)
	≥75 years	74.43 (n=14)

EQ5D-VAS: EuroQol 5-Dimensions visual analogue scale

Table S5: Multivariate regression model 3 - EQ5D5L

Characteristic	Beta	95% CI[1]	p-value
(Intercept)	91	84, 99	<0.001
DCSI	-1.5	-2.0, -1.0	<0.001
Score EPICES	-0.23	-0.28, -0.18	<0.001
Sex			
female	—	—	
male	5.0	3.5, 6.6	<0.001
Age (years)	0.00	-0.09, 0.09	>0.9
Diabetes duration (years)	-0.04	-0.13, 0.05	0.4
Insulin treatment			
Multi daily injection	—	—	
Pump	0.31	-1.4, 2.0	0.7
Hypoglycemia minimizer	2.5	-1.4, 6.4	0.2
Automated insulin delivery systems	0.94	-1.7, 3.6	0.5
HbA1c (%)	-1.5	-2.2, -0.83	<0.001
Tobacco			
no	—	—	

Characteristic	Beta	95% CI[1]	p-value
yes	-1.6	-3.2, -0.03	0.046
BMI (kg/m²)	-0.14	-0.30, 0.02	0.089
Physical activity (MET/week)	0.00	0.00, 0.00	0.2

[1]CI = Confidence Interval

Table S6: ADDQoL: subdomains of HRQoL

Characteristic	N	N = 1,375[1]
leisure activities	1,375	-3.10 ±2.66
working life	1,098	-2.99 ±2.86
journeys	1,375	-2.73 ±2.65
holidays	1,321	-3.70 ±2.85
physical health	1,375	-3.75 ±2.84
family life	1,346	-2.13 ±2.76
friendship and social life	1,375	-2.11 ±2.56
personal relationship	1,284	-2.60 ±2.92
sex life	1,298	-2.54 ±2.74
physical appearance	1,375	-2.25 ±2.69
self-confidence	1,375	-2.45 ±3.07
motivation	1,375	-2.19 ±2.97
people's reactions	1,375	-0.97 ±1.99
feelings about future	1,375	-3.58 ±3.00
financial situation	1,375	-1.10 ±2.22
living conditions	1,375	-2.07 ±2.69
dependence on others	1,375	-2.45 ±2.97
freedom to eat	1,375	-3.77 ±3.17
freedom to drink	1,375	-3.00 ±3.09
**ADDQoL average-weighted impact score**	**1,375**	**-2.60 ±1.74**

[1]Mean ±SD

ADDQoL: Audit of Diabetes-Dependent Quality of Life

Figure S1: ADDQoL: subdomains of HRQoL

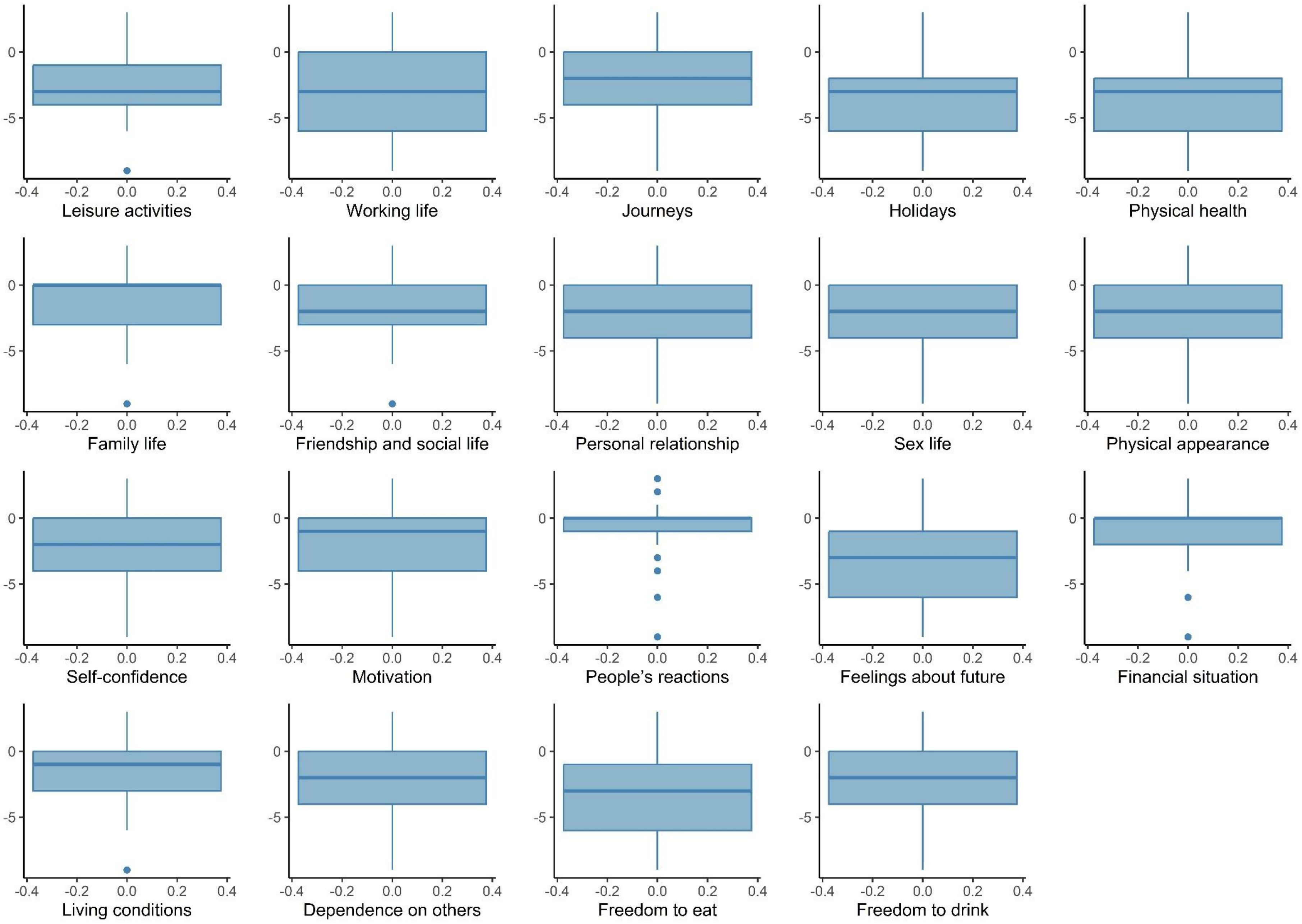

Supplement: Supplementary file 1 — Data S1. Supporting Information. [file DOM-28-1201-s001.pdf]
